# Nonlinear Regression Operating on Microstructures Described from Topological Data Analysis for the Real-Time Prediction of Effective Properties

**DOI:** 10.3390/ma13102335

**Published:** 2020-05-19

**Authors:** Minyoung Yun, Clara Argerich, Elias Cueto, Jean Louis Duval, Francisco Chinesta

**Affiliations:** 1 PIMM Laboratory & ESI Group Chair, Arts et Métiers Institute of Technology, CNRS, Cnam, HESAM Université, 151 boulevard de l’Hôpital, 75013 Paris, France; minyoung.yun@ensam.eu (M.Y.); clara.argerich_martin@ensam.eu (C.A.); 2 Aragon Institute of Engineering Research, Universidad de Zaragoza, 50009 Zaragoza, Spain; ecueto@unizar.es; 3 ESI Group, Bâtiment Seville, 3bis rue Saarinen, 50468 Rungis, France; Jean-Louis.Duval@esi-group.com

**Keywords:** machine learning, data-driven mechanics, TDA, *Code2Vect*, nonlinear regression, effective properties, microstructures

## Abstract

Real-time decision making needs evaluating quantities of interest (QoI) in almost real time. When these QoI are related to models based on physics, the use of Model Order Reduction techniques allows speeding-up calculations, enabling fast and accurate evaluations. To accommodate real-time constraints, a valuable route consists of computing parametric solutions—the so-called computational vademecums—that constructed off-line, can be inspected on-line. However, when dealing with shapes and topologies (complex or rich microstructures) their parametric description constitutes a major difficulty. In this paper, we propose using Topological Data Analysis for describing those rich topologies and morphologies in a concise way, and then using the associated topological descriptions for generating accurate supervised classification and nonlinear regression, enabling an almost real-time evaluation of QoI and the associated decision making.

## 1. Introduction

Recently, industry is experiencing a new revolution. In the past, product design, as well as their associated manufacturing processes, were based on the use of nominal models, nominal loadings (in their broadest sense), and a small amount of data for calibrating those models, with the product performance as a design target.

Very recently, predictions enabling real-time decision-making targeting zero defects in processing and zero unexpected faults in operation, were needed everywhere within the Internet of Things (IoT) paradigm, on the work-floor (smart processes), in the city (autonomous systems and smart-city), at the nation level (e.g., smart nation), etc., i.e., anywhere where engineering designs operate.

In those circumstances, the use of traditional simulation-based engineering (SBE) that was the major protagonist of 20th century engineering, is not anymore a valuable option due to three main reasons: (i) models become sometimes crude approximations of the observed reality; (ii) assimilating data enabling the continuous calibration of the models in operation remains difficult to perform under the stringent real-time constraint; and (iii) the real-time simulation of those extremely complex mathematical models needs alternative techniques to those commonly employed in traditional SBE.

It was at the beginning of the XXI century that two new revolutions in the domain of digital engineering emerged.

### 1.1. Model Order Reduction

Advances in applied mathematics, computer science (high-performance computing) and computational mechanics met to give rise to a diversity of Model Order Reduction (MOR) techniques [1]. These techniques do not reduce or modify the model, they simply reduce the complexity of its resolution and thus transform a complex and time-consuming calculation, into a real-time response while maintaining precision. These new techniques have completely altered traditional approaches of simulation, optimization, inverse analysis, control and uncertainty propagation, all them operating under the stringent real-time constraint.

In a few words, when approximating the solution u(x,t) of a given Partial Differential Equation (PDE), the multipurpose finite element method assumes an approximation

(1) $$u\left(x,t\right)=\sum_{i=1}^{N}U_{i}\left(t\right)N_{i}\left(x\right),$$

where $U_{i}$ represents the value of the unknown field at node *i* and $N_{i}\left(x\right)$ is the associated shape function. When N (the number of nodes) increases the solution process becomes cumbersome.

POD-based model order reduction learns offline the most adequate (in a given sense) reduced approximation basis $\left\{\phi_{1}\left(x\right),\cdots ,\phi_{R}\left(x\right)\right\}$, and project the solution in it

(2) $$u\left(x,t\right)\approx \sum_{i=1}^{R}\xi_{i}\left(t\right)\phi_{i}\left(x\right),$$

where now, the complexity scales with R instead of N, with R≪N in general.

The so-called Proper Generalized Decomposition (PGD from now on) goes a step forward and assume a general approximation

(3) $$u\left(x,t\right)\approx \sum_{i=1}^{M}T_{i}\left(t\right)X_{i}\left(x\right),$$

where now both the space and time functions, $X_{i}\left(x\right)$ and $T_{i}\left(t\right)$ respectively, are computed during the solution process.

A particularly appealing extension of the just introduced space-time separated representation consists of the space-time-parameter separated representation leading to the a so-called *computational vademecum* that expresses the solution of a parametrized PDE from [2,3]

(4) $$u\left(x,t,\mu_{1},\ldots ,\mu_{Q}\right)\approx \sum_{i=1}^{M}X_{i}\left(x\right)T_{i}\left(t\right)\prod_{j=1}^{Q}M_{i}^{j}\left(\mu_{j}\right),$$

where $\mu_{j}$, j=1,⋯,Q, represent the model parameters. Once constructed off-line that parametric solution (4), it offers under very stringent real-time constraints—in the order of milliseconds—simulation, optimization, inverse analysis, uncertainty propagation and simulation-based control, to cite a few. Thus, at the beginning of the third millennium a real-time dialogue with physics no longer seemed to be the domain of the impossible.

PGD-based techniques have been widely considered for the real-time simulation and decision-making in a variety of problems of industrial relevance. However, prior to use it, one must extract the parameters to be included as extra-coordinates in the problem statement, and then included in the parametric representation of its solution. In the case of morphological and topological descriptions, as considered later in the present work, the extraction of the adequate parametrization represents the most difficult task. Some attempts of combing PGD-based MOR and manifold learning [4] were addressed in [5,6,7,8].

### 1.2. Engineered Artificial Intelligence

Data bursts within engineering disciplines. For years, data was used in other areas where models were less developed or remained quite inaccurate. Data collected massively was successfully classified, cured, distilled, … using artificial intelligence (AI) techniques. Thus, correlations between data ca be removed, proving that a certain simplicity remains hidden behind a rather apparent complexity. Data-driven modeling developed exponentially and advanced artificial intelligence techniques were developed, covering six major domains: (i) Multidimensional data visualization [9]; (ii) Data classification and clustering [10,11]; (iii) Learning models from input/output pairs of data, with adequate techniques enabling real-time learning and able to operate in the low-data limit (e.g., sPGD [12], *Code2Vect* [13], iDMD [14,15,16], NN [17], ThemodynML [5,18], …); (iv) Knowledge extraction in order to identifying combined parameters and model richness/complexity, discovering hidden parameters, discarding useless parameters or even to extract governing equations; (v) Explaining for certifying; and (vi) Hybridizing physics and data for defining advanced and powerful Dynamic Data-Driven Application Systems, DDDAS [19].

However, these data-driven models, when used in engineering and industry, were quickly confronted with three major and recurrent difficulties: (i) the need for a huge amount of data to make predictions accurate and reliable, knowing that data is synonymous with cost (acquisition and processing costs); (ii) the difficulty of explaining and interpreting predictions obtained by artificial intelligence; and (iii) related to the the latter, the difficulty of certifying engineering products.

### 1.3. Towards Real-Time Decision Making

In summary, on one side models based on physics can be solved fast but, as discussed, in many engineering areas they remain poor approximations of the real components and systems. On the other hand, when approaching the problem from the data perspective, impressive amounts of data are sometimes needed (with the associated cost and technological difficulty of collecting them), to be processed in real time and then explained in order to certify both the designs and the decisions.

A possible winning option consists of merging both concepts and methodologies. The *hybrid paradigm* was born [19,20], associating in it two type of models: the first based on physics; the second being a completely new type of model, more pragmatic and phenomenological, based on data.

Real-time decision making in engineering design, manufacturing and predictive and operational maintenance, needs the evaluation of quantities of interest in almost real-time. The present work aims at proposing a technique able to determine under the stringent real-time constraints, effective properties of a complex microstructure by assimilating an image of it.

To conciliate accuracy and real-time constraints, the hybrid paradigm is retained: (i) the prediction engine will be trained offline from data coming form physics; then (ii) a non-linear regression, acting on some topological descriptors extracted from those images, will ensure a real-time evaluation of the effective properties (in the present case the homogenized thermal conductivity).

As previously discussed, dealing with shapes and topologies, the parametric description requires performant techniques able to express them in a compact and concise way. In this paper, we propose using Topological Data Analysis, TDA [21], for representing these rich topologies and morphologies, and then using the associated topological descriptors for generating accurate supervised classification and nonlinear regressions, enabling an almost real-time evaluation of the quantities of interest.

In the next section we will present the main methodologies used in the present study, that will be considered later for the training and then for the real-time evaluation of effective properties (homogenized thermal conductivity) of rich microstructures.

## 2. Methods

This section revisits the main methodologies that will be considered later for real-time classification and prediction of the effective thermal properties from collected images. For that purpose we will consider a rich enough training stage that consists of generating several microstructures whose effective thermal conductivity will be evaluated by using a standard linear homogenization technique, revisited in Section 2.1.

In order to associate the resulting homogenized conductivity tensor to each microstructure, the last must be described in a compact and concise way. For that purpose Topological Data Analysis and Principal Component Analysis (PCA) will be employed. Both are revisited in Section 2.2 and Section 2.3, respectively.

The last step aims at performing a nonlinear regression to link the parameters extracted by the TDA to the thermal conductivity. The technique retained in our study is the so-called *Code2Vect* nonlinear regression, revisited in Section 2.4.

### 2.1. Linear Homogenization Procedure

Due to the microscopic nature of heterogeneity, a procedure is required for extracting the effective thermal conductivity. In what follows we proceed in the linear case, as was also the case in [22], in a representative volume element Ω with a microstructure perfectly defined at that scale. The microscopic conductivity k(x) is known at every point x∈Ω.

The macroscopic temperature gradient G is defined from the space average

(5) $$G\;=\;\langle g\left(x\right)\rangle \equiv \frac{1}{\left|\Omega \right|}\int_{\Omega }g\left(x\right)\;dx,$$

where g(x) represents the microscopic temperature gradient, i.e., g(x)=∇T(x).

We define the localization tensor L(x) such that

(6) g(x)=L(x)G.

The microscopic heat flux q(x) follows the Fourier law

(7) q(x)=−k(x)g(x),

and its macroscopic counterpart Q reads

(8) Q=〈q(x)〉=〈−k(x)g(x)〉=〈−k(x)L(x)〉G,

from which the homogenized thermal conductivity reads

(9) K=〈−k(x)L(x)〉.

Thus, the calculation of the homogenized thermal conductivity tensor only needs the computation of the tensor L(x). The present work considers the simplest procedure that in the 2D case consists of solving two steady state thermal problems in Ω

(10) $$\left\{\begin{array}{c} \nabla \cdot \left(k\left(x\right)\;\nabla T^{1}\left(x\right)\right)\;=\;0\\ T^{1}\left(x\in \partial \Omega \right)=x, \end{array}\right.,$$

and

(11) $$\left\{\begin{array}{c} \nabla \cdot \left(k\left(x\right)\;\nabla T^{2}\left(x\right)\right)\;=\;0\\ T^{2}\left(x\in \partial \Omega \right)=y \end{array}\right.,$$

whose solutions verify by construction

(12) $$\left\{\begin{array}{c} G^{1}\;=\;{\langle \nabla T^{1}\left(x\right)\rangle }^{T}\;=\;\left(1,0\right)\\ G^{2}\;=\;{\langle \nabla T^{2}\left(x\right)\rangle }^{T}\;=\;\left(0,1\right) \end{array}\right.,$$

and whose gradients define the localization tensor columns

(13) $$L\left(x\right)\;=\;\left(\nabla T^{1}\left(x\right)\;\;\;\nabla T^{2}\left(x\right)\right),$$

that allows calculating the effective thermal conductivity.

**Remark** **1.** *In the present work, since we are only interested in the effective thermal conduction along the y-direction, a single problem, problem (11), suffices for calculating the only component of interest, component $K_{22}$.*

### 2.2. Topological Data Analysis

Topological data analysis, TDA [21], is one of the most promising techniques in high-dimensional data analysis. In essence, TDA is a powerful tool to find the topology of data: if there are clusters, a manifold structure or even noise that is not relevant for the analysis.

**Figure 1:**
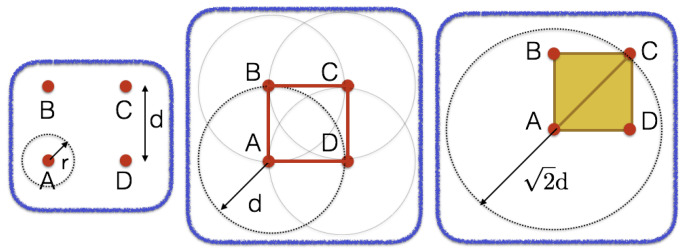
Illustrating TDA: (**left**) For r<d the four points (A, B, C and D) remain disconnected; (**center**) At r=d the hole ABCD appear from the four edges AB, BC, CD and DA; (**right**) The just created hole persist until $r=\sqrt{2}d$, value at which A connects with C and the two resulting triangles ABC and ACD cover the initial hole that disappears consequently.

For an intuitive description of the method consider the set of points depicted in Figure 1. In general, these points will live in high dimensional spaces, such that their intrinsic topology will not be visible at first glance. We then equip the set with a distance parameter *r*. By making *r* grow, different *k*-simplexes will appear. Remember that a 0-simplex is a point, a 1-simplex is an edge, a 2-simplex is a triangle, on so on.

As *r* grows, holes appear (as the one defined by the edges between points A, B, C and D in Figure 1, for instance when r=d), and disappear for higher values of *r* (when $r=\sqrt{2}d$, the initial hole is covered by triangles ABC and ACD). Which is important in this discussion is that the overall structure of data is the one that *persists* for longer *r* values. Holes defined by noisy data are rapidly eliminated from the simple complex.

The value of *r* at which a hole appears, and then the one at which it disappears, defines a bar joining both, which characterizes the hole persistence. When collecting all the bars associated with all the holes appearing and then disappearing when *r* grows, the so-called persistence barcode results, the last representing compactly a given morphology.

An alternative consists of using a 2D representation, the so-called persistence diagram (PD), reporting in the $x_{1}$-axis the value of *r* at which a hole appears, and on the $x_{2}$-axis the value at which it disappears. Obviously, with the hole birth preceding its death, all the point are place on the upper domain defined by the bisector $x_{2}=x_{1}$, and any point $\left(x_{1},x_{2}\right)$ remaining close to that bisector represents noise, a small scale, with the associated hole death following immediately its birth. Points far from the bisector represent the topology that persists.

The persistence barcode and the persistence diagram are two representations with a high physical content; however both representations can not be used for comparison purposes, because they are defined in a non-metric space where the calculation of distances for concluding on proximity has not sense.

To move to a more appropriate space making possible the calculation of distances, we first transform the persistence diagram according to $\left(x_{1},x_{2}\right)\to \left(y_{1}=x_{1},y_{2}=x_{2}-x_{1}\right)$ and then apply on the last a convolution (usually with a Gaussian kernel) leading to the so-called persistence image (PI) y, the last defined in a vector space, $y\in R^{D}$, that allows applying most of AI algorithms [23].

### 2.3. Principal Component Analysis

TDA is able to analyze a complex microstructure through its associated image, and to extract its relevant topological features in form of a persistence image, that can be viewed a matrix. However, using these matrix components is not the most compact and concise way of representing the microstructure, because it contains too many components that makes difficult using it for constructing regressions. Thus, in practice, a linear dimensionality reduction such as principal component analysis (PCA) can be applied for extracting the most representative modes of the persistence images and then to represent in a compact and concise way the microstructures by using the weight associated with the most important modes extracted.

Let us consider a vector $y\in R^{D}$ containing the different components of a persistence image. When considering a set of P microstructures, the associated PIs lead to $y_{i}$, i=1,⋯,P. If they are somehow correlated, there will be a linear transformation W defining the vector $\xi \in R^{d}$, with d<D, which contains the still unknown *latent variables*, such that [4]

(14) y=Wξ.

The transformation matrix W, D×d, satisfies the orthogonality condition $W^{T}W=I_{d}$, where $I_{d}$ represents the d×d identity matrix.

PCA proceeds by guaranteeing maximal preserved variance and de-correlation in the latent variable set ξ. Thus, the covariance matrix of ξ,

(15) $$C_{\xi \xi }=E\left\{\Xi \Xi {}^{T}\right\},$$

will be diagonal. PCA will then extract the *d* uncorrelated latent variables from

(16) $$C_{yy}=E\left\{YY^{T}\right\}=E\left\{W\Xi \Xi {}^{T}W^{T}\right\}=WE\left\{\Xi \Xi {}^{T}\right\}W^{T}=WC_{\xi \xi }W^{T},$$

that pre- and post-multiplying by $W^{T}$ and W, respectively, reads

(17) $$C_{\xi \xi }=W^{T}C_{yy}W.$$

By factorizing the covariance matrix $C_{yy}$, applying the singular value decomposition, SVD,

(18) $$C_{yy}=V\Lambda V^{T},$$

and taking into account Equation (17), it results

(19) $$C_{\xi \xi }=W^{T}V\Lambda V^{T}W,$$

that holds when the *d* columns of W are taken collinear with *d* columns of V, i.e.,

(20) $$W=VI_{D\times d}.$$

### 2.4. Code2Vect

**Figure 2:**
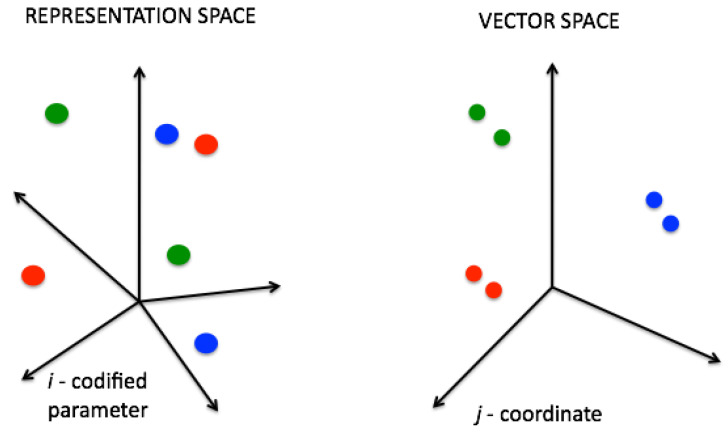
Input space ξ (**left**) and target vector space z (**right**).

*Code2Vect* [13] maps data into a vector space where the distance between points is proportional to the difference of the QoI associated with those points, as sketched in Figure 2.

We assume the available data consisting of P *d*-dimensional arrays, $\xi_{i}\in R^{d}$, with a QoI $O_{i}$ associated with each datum. The images, $z_{i}\in R^{q}$ (q=2 in our numerical implementation for the sake of visualization clarity), results from

(21) $$z_{i}=W\xi_{i},\;\;\;i=1,\cdots ,P,$$

that preserves the quantity of interest associated with is origin point $\xi_{i}$, denoted by $O_{i}$.

In order to place points such that distances scales with their QoI differences we enforce

(22) $$\left(W\left(\xi_{i}-\xi_{j}\right)\right)\cdot W\left(\xi_{i}-\xi_{j}\right))={∥z_{i}-z_{j}∥}^{2}=\left|O_{i}-O_{j}\right|.$$

Thus, there are $\frac{P^{2}}{2}-P$ relations to determine the q×d+P×q unknowns. Linear mappings are limited and do not allow proceeding in nonlinear settings. Thus, a better choice consists of a nonlinear mapping W(ξ), expressible as a general polynomial form.

## 3. Results

### 3.1. Model Training

**Figure 3:**
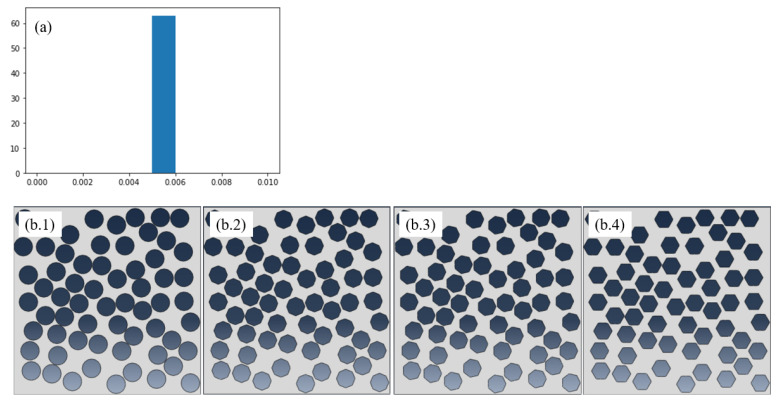
(**a**) Histogram of pores radius; (**b**) Pore shapes: Circle, Octagon, Heptagon and Hexagon.

Several microstructures, based on a population of holes (from now on called pores) with different sizes, shapes, location, and number of pores, distributed in the 2D square domain Ω, are created. Four of these microstructures are shown in Figure 3. They are equipped with a mesh on which finite element calculations will be done for computing the reference effective (homogenized) thermal conductivity, in particular the component $K_{22}$ of the homogenized conductivity tensor.

These meshes also serve to apply the TDA in order to obtain the persistence diagram (PD) and its associated persistence image (PI). As previously indicated, the last consists of a convolution applied on the former. Each persistence image defines a 20×20 matrix, or its vector counterpart $y_{i}\in R^{400}$.

Thus, TDA is able to analyze a complex microstructure through its image, and extract its relevant topological features in form of a persistence image, that can be viewed as a matrix. However, this matrix still contains too much information (its number of components, here 20×20) to perform classification or regression when not too much data is available (scarce-data limit). Obviously, large amounts of synthetic data can be produced by solving numerically thousands or even millions of thermal problems. However, in engineering cheap solutions are usually preferred, and in particular smart-data is preferred to its big counterpart. Efficiency seems a better option that brute force, and for this reason, here we prefer keeping the amount of data as reduced as possible, and compensate its absence by enhancing the amount of information that data contains.

Thus, persistence images are still not the most compact way of representing the topological and morphological features of the analyzed microstructures. For improving the representation we apply a linear dimensionality reduction, the principal component analysis, for extracting the most representative modes of the persistence images. Thus, the weights of those PCA modes will constitute the compact and concise way to represent those microstructures.

From a practical viewpoint PCA allowed reducing from 400=(20×20) the dimension of PI resulting from TDA, to 3 dimensions. Thus, each analyzed microstructure is concisely represented by 3 coordinates (the weights of the first three most relevant PCA modes) and each one has attached a QoI, the effective thermal conductivity $K_{22}$ obtained from a finite element simulation following the rationale described in Section 2.1. Now, the nonlinear regression relating the output, the QoI (the effective thermal conductivity in our case), with the parameters describing the microstructure, the three PCA weights, is performed by applying the *Code2Vect* nonlinear regression, summarized in Section 2.4.

As soon as the regression is constructed at the present training stage, it could be used online for predicting the conductivity of new microstructures.

### 3.2. Inferring Effective Properties

We prepared 5 samples, four of them were used in the training stage, represented in Figure 3, in which the pores volume fraction was kept constant (ϕ=0.5) and the spatial distribution almost uniform.

**Figure 4:**
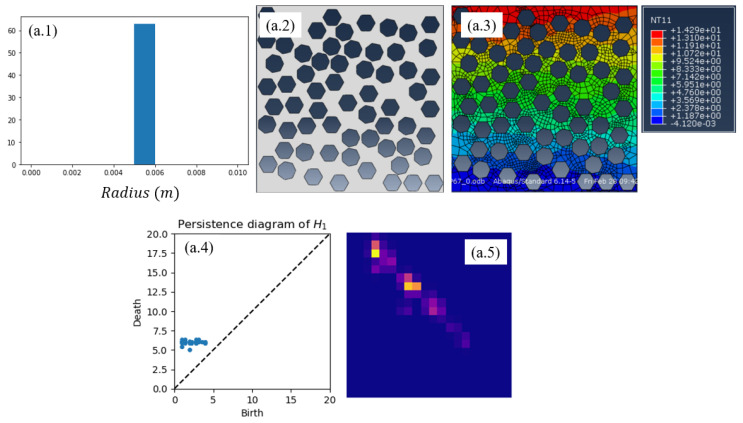
(**a.1**) Histogram of the pores radius; (**a.2**) considered microstructure; (**a.3**) temperature field used for computing the effective thermal conductivity that will serve as reference for evaluating the regression performance; (**a.4**) persistence diagram; and (**a.5**) persistence image.

The constructed nonlinear regression (based on the use of *Code2Vect*) described in the previous section, is now applied to the sample shown in Figure 4 where while keeping the same almost uniform pore distribution and the same volume fraction, hexagons and heptagons were randomly mixed. In this same figure, the solution of the thermal problem at the microscopic scale for obtaining the effective thermal conductivity that will serve as reference value, is also included. Finally, it also shows both the PD and the PI.

The PI, $y\in R^{D}$, is then projected into the three retained orthonormal PCA modes to give the thee weights that constitute the data $\xi \in R^{3}$ (d=3) to be processed by the nonlinear regression (based on the *Code2Vect*) that produces vector $z\in R^{2}$ (we enforce a 2D representation, q=2, for the sake of clarity in the data visualization)

(23) z=W(ξ)ξ,

and then identify the set S(z) of data $z_{i}$ closest to z, from which the QoI, the effective thermal conductivity, is interpolated

(24) $$O=\sum_{i\in S(z)}F\left(z,z_{i}\right)\;O_{i},$$

with in the present case $O\equiv K_{22}$ and with radial bases as interpolation functions $F(z,z_{i})$.

**Figure 5:**
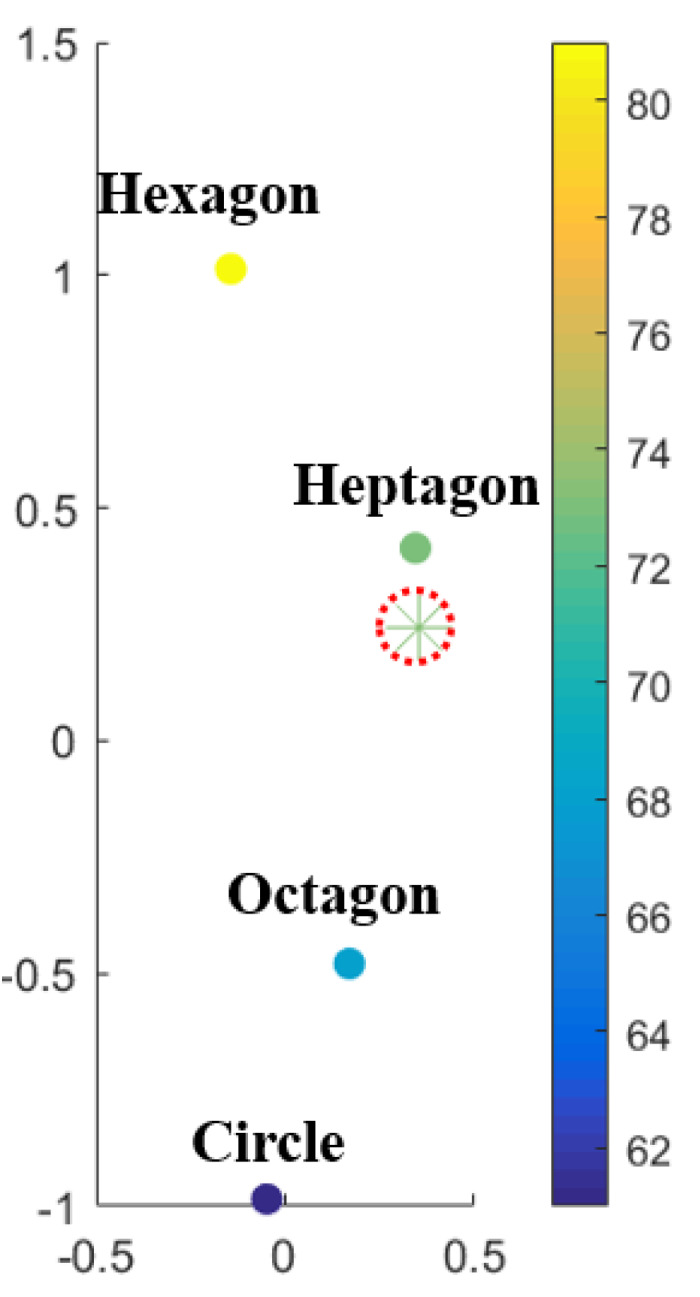
Interpolation space z with color scaling with the values of the effective thermal conductivity $K_{22}$.

Figure 5 places z with respect to its neighbors, where color scales with the target quantity, that is, with $K_{22}$. The inferred value of the effective thermal conductivity $K_{22}$ using Equation (24) for the micorstructure depcited in Figure 4 results $K_{22}\left(z\right)=73.4$ W/mK, very close to the reference value computed numerically from the temperature distribution shown also in Figure 4, of $K_{22,\mathrm{REF}}=74$ W/mK.

### 3.3. Microstructures with Varying Shapes and Size Distribution

**Figure 6:**
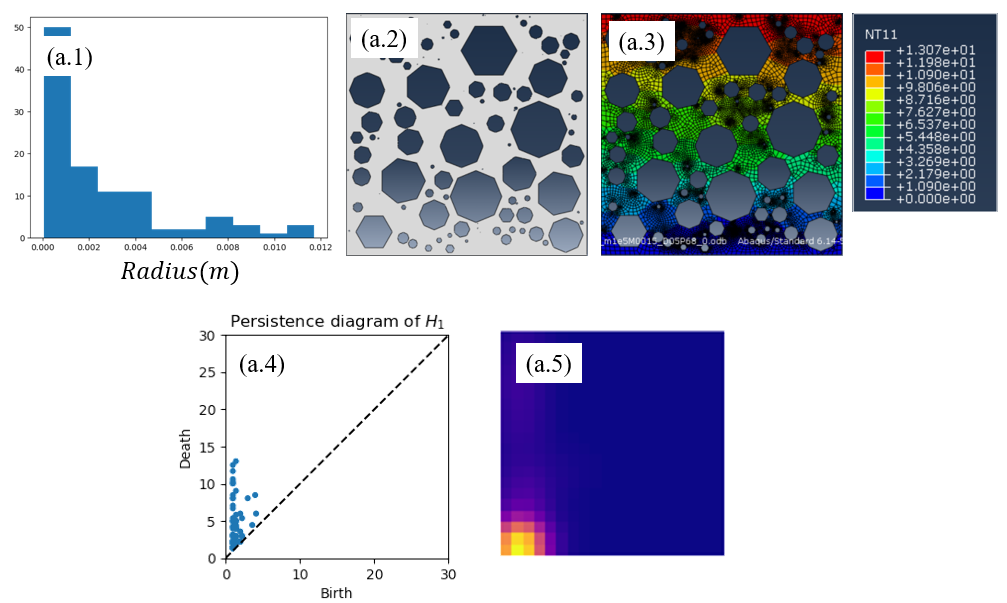
(**a.1**) Histogram of pores radius; (**a.2**) testing microstructure; (**a.3**) temperature field used for calculating the reference effective thermal conductivity; (**a.4**) persistence diagram; and (**a.5**) persistence image.

**Figure 7:**
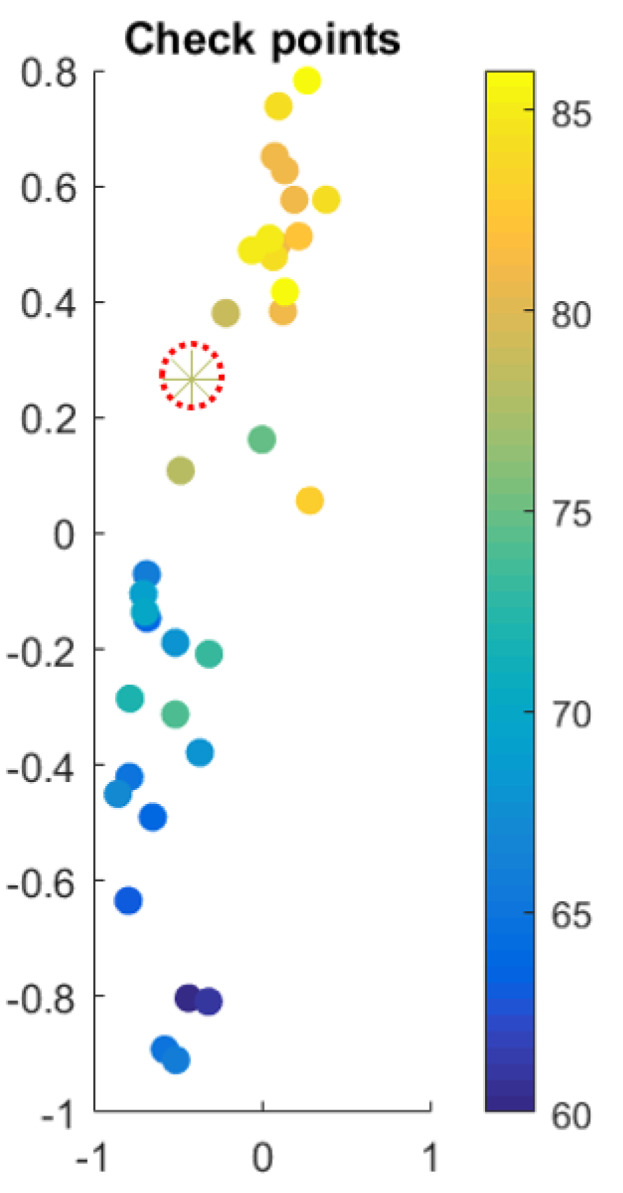
Interpolation space z with color scaling with the values of the effective thermal conductivity $K_{22}$.

These first preliminary successful results were pushed forward by considering quite more complex microstructures. Thus, a total of 35 samples were generated, while varying other parameters, in particular pore size (following uniform and Gamma distributions) and pore shape (circular or 5 to 8 side polygons, randomly chosen). The volume fraction was kept constant (ϕ=0.5). 34 samples were used in the training, keeping one, the one shown in Figure 6, for inferring the thermal conductivity and concluding on the ability of the proposed technique to infer accurately it. Figure 7 places the considered microstructure in the z-space where the thermal conductivity is interpolated, to infer the value of $K_{22}=81$ W/mK, for a reference value of $K_{22,\mathrm{REF}}=78$ W/mK.

To check the prediction improvement with the sampling richness, the effective thermal conductivity in the microstructure shown in Figure 6 while considering different samplings in the training stage, from 13 to 35 microstructures, with the relative errors reported in Table 1.

## 4. Conclusions

The present study proves that effective properties can be associated with microstructures with complex morphological and topological features. For this purpose, those features are extracted by using TDA, post-compressed by using linear dimensionality reduction (PCA) which output represents the parameters employed by the nonlinear *Code2Vect* regression that finally assign a effective property (here the effective thermal conductivity) to a given microstructure.

The procedure demonstrated its robustness and performance in the low-data limit, as well as its capacity to provide better predictions when considering larger training sets. It successfully combines physics-based data for learning purposes, with almost real-time inference based on the topological analysis of images.

## Figures and Tables

**Table 1:** Relative error in the effective conductivity prediction depending on the number of samples considered in the regression (training stage).

Number of Samples	Relative Error
13	0.076
16	0.056
19	0.046
35	0.037
